# Individual Differences in Infants’ Curiosity Are Linked to Cognitive Capacity in Early Childhood

**DOI:** 10.1111/desc.70090

**Published:** 2025-11-07

**Authors:** Eline R. de Boer, Francesco Poli, Marlene Meyer, Rogier B. Mars, Sabine Hunnius

**Affiliations:** ^1^ Donders Institute for Brain, Cognition and Behaviour Radboud University Nijmegen The Netherlands; ^2^ MRC Cognition and Brain Sciences Unit University of Cambridge Cambridge UK; ^3^ Wellcome Centre for Integrative Neuroimaging, Centre for Functional MRI of the Brain (FMRIB), Nuffield Department of Clinical Neurosciences, John Radcliffe Hospital University of Oxford Oxford UK

**Keywords:** childhood intelligence, cognitive development, infant curiosity, information gain, IQ, longitudinal

## Abstract

Research has shown that infants are curious and actively seek situations from which they can learn. For instance, a recent eye‐tracking study demonstrates that babies tend to allocate their attention to stimuli that offer opportunities for learning new information. Interestingly, however, the degree to which attention is guided by information gain varies among individual infants. This longitudinal study provides the first empirical evidence suggesting that these early individual differences in infants’ sensitivity to information gain are linked to later cognitive development. Specifically, we found that the extent to which infants’ attention was guided by information gain at 8 months was related to their IQ scores at 3.5 years of age (*n* = 60, 50% female): especially children who displayed the greatest curiosity as infants tended to have a more favourable cognitive development. These findings demonstrate the lasting consequences of early existing differences in curiosity‐driven exploration for later childhood cognitive development.

**Summary:**

- We link individual differences in curiosity, measured as infants’ sensitivity to information gain, to later cognitive outcomes.
- Infants’ sensitivity to information gain was related to their IQ scores 3 years later.
- Curiosity may act as a boost, improving cognitive functioning for those children that were especially curious during infancy.

## 1 Introduction

Infants are extraordinarily quick learners who swiftly acquire knowledge about the world. Understanding the attentional mechanisms underlying infants' rapid learning and the role they play for later cognitive functioning has been a core focus of developmental psychology and cognitive sciences for decades (e.g., Fantz 1964; Kavšek 2004; Rose and Feldman 1997). Previous research into these attentional mechanisms has revealed that infants exhibit a bias towards stimuli that are highly novel (e.g., Fantz 1964), surprising (e.g., Baillargeon et al. 1985; Stahl and Feigenson 2015), or intermediately complex (Kidd et al. 2012). These studies measured infants’ visual attention through their looking behaviour, demonstrating that infants prefer to look at novel (over familiar) stimuli, events that violate their expectations, and stimuli that are neither too simple nor too complex. They also show that by focusing their attention on such stimulus characteristics (such as toys defying solidity or gravity), infants learn effectively (e.g., Stahl and Feigenson 2015).

However, these biases alone are not sufficient to support efficient learning (Bazhydai et al. 2019; Chu and Schulz 2020; Oudeyer 2018). For instance, not all novel information is meaningful (Dubey and Griffiths 2020; Gottlieb et al. 2013). And if children focused solely on the most surprising events, they would risk getting stuck in exploring stimuli that are random and unlearnable, like the pattern of raindrops falling on the ground (Chu and Schulz 2020; Gottlieb et al. 2013). Also, while a focus on intermediate complexity offers a more nuanced perspective on children's (visual) exploration, it may overlook the basic human drive to attend to novel events that might appear highly complex at first (Dubey and Griffiths 2020). Furthermore, determining the optimal level of complexity is rather arbitrary and likely varies across individuals—some children may perceive a stimulus as more complex than others—and likely changes over time as children gain more experience with a particular event.

Recent research on curiosity offers an alternative explanation for why infants learn so much, so quickly. The learning progress hypothesis proposes that learning itself is intrinsically rewarding and thereby serves as a key element of humans' motivation to explore and seek out information (Oudeyer et al. 2016; Ten et al. 2021). According to this hypothesis, infants are sensitive to the amount of information they can gain from an environment, driving their attention to aspects of the environment they can learn from—a mechanism identified as a key aspect of curiosity (Baer and Kidd 2022; Bazhydai et al. 2019; Kidd and Hayden 2015; Oudeyer et al. 2016; Poli, O'Reilly, et al. 2024; Ten et al. 2021). The hypothesis suggests that individuals are drawn to stimuli and activities just beyond their current knowledge level, which helps them avoid getting stuck in environments that are overly simple or too complex, as well as those that are novel but not learnable (Oudeyer et al. 2016; Poli et al. 2020). Like the intermediate complexity account, the Learning Progress hypothesis points to the richest learning opportunities lying just beyond what the child currently knows or can do (Andersen et al. 2022; Berlyne 1966; Oudeyer et al. 2007; Piaget 1952; Vygotsky and Cole 1978). Yet, the learning progress approach models curiosity as a dynamic and self‐updating process and does not focus on the complexity of the features of a specific stimulus or event, but rather on the learning itself.

**FIGURE 1:**
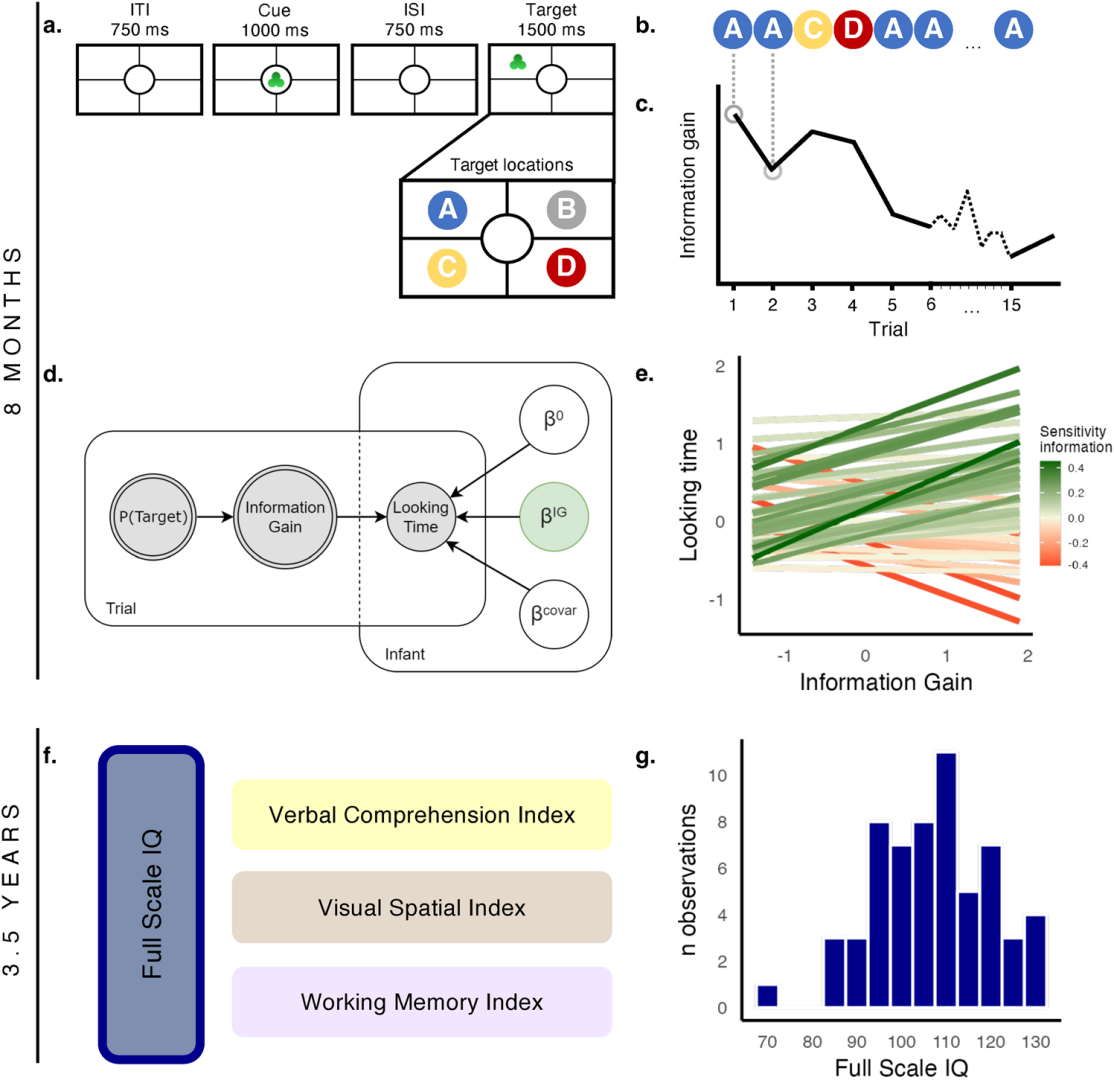
Study outline. *Note*: (a) Infants were shown multiple sequences of cue‐target trials, with each sequence featuring a different stimulus shape (in this example, a trefoil). The cue first appeared in the centre of the screen and then was followed by an identical target stimulus in one of four possible locations (i.e., A–D). (b) The probabilistic structure of each sequence was statistically manipulated so that the target was more likely to appear in one location (in this example, location A) than in the others. (c) This resulted in each target stimulus providing a different amount of information about the sequence's probabilistic structure. This measure of information gain was quantified using KL divergence. (d) A hierarchical Bayesian model assessed infants’ sensitivity to information by relating their looking time to the target stimulus with the information gain provided by the stimulus. (e) Overall, infants’ looking times were modulated by the information gain offered by the stimuli. However, individual infants displayed varying degrees of sensitivity to information (i.e., different slopes). A stronger positive relation indicates that infants tailored their looking time more to the available information gain, reflecting more curiosity‐driven learning. (f) At 3.5 years of age, participants' cognitive abilities were assessed using the standardized WPPSI‐IV‐NL intelligence test, which provides a general intelligence factor (Full Scale IQ) along with three subindices. (g) The Full Scale IQ that was used as our main outcome measure showed sufficient spread (*N* = 60).

Recent empirical findings provide support for this idea. Several studies have shown that infants are sensitive to whether stimuli are informative (Addyman and Mareschal 2013; Bazhydai et al. 2020; Kidd et al. 2012; Ruggeri et al. 2019; Twomey and Westermann 2018) and sample their surroundings to maximize the amount of information they can gain (Poli et al. 2020). In their study, Poli and colleagues (2020) presented infants with a screen‐based visual learning task that contained a series of cue‐target sequences (Figure 1a). For each trial, they quantified the level of surprise of the stimulus and the overall predictability of the environment, while also tracking the *information gain*, the amount of information each trial provided (Figure 1b,c). Their key objective was to determine which information‐theoretic measure guides infants’ attention most. Their results revealed that information gain was the strongest predictor of infants’ looking behaviour. Infants spent more time looking at stimuli when there was more information to be gained, and they tended to look away when the stimuli no longer offered new information. The level of predictability and surprise of the stimuli also guided infants’ looking behaviour, but to a lesser extent.

Interestingly, subsequent experimental and computational work has shown that there is substantial variability in the degree to which infants are sensitive to the informativeness of stimuli (Poli, Ghilardi, et al. 2024). Some infants display a greater inclination to attend to new information than others, as evidenced by their prolonged looking towards information‐rich compared to information‐poor stimuli (Figure 1e). In other words, some infants appear to be more curious than others. This raises the question of whether these individual variations in infants’ sensitivity to information are predictive of their later cognitive functioning. It seems plausible that children who are more focused on the informative aspects of their environment, acquire more knowledge through the years, resulting in enhanced cognitive functioning (Chen et al. 2022; Muentener et al. 2018; Shah et al. 2018, 2023; Stahl and Feigenson 2015). Yet, empirical evidence linking such individual differences in infants’ sensitivity to information with later cognitive functioning is still lacking.

In the past, individual differences in early looking behaviour have been related to later cognitive functioning (e.g., Bornstein and Sigman 1986; Cuevas and Bell 2014; Dougherty and Haith 1997; Hitzert et al. 2014; Kavšek 2004; Rose and Wallace 1985). So far, one of the most reliable predictors in this domain has been infants’ habituation (Bornstein et al. 2013; Kavšek, 2004). It has even been suggested that infants’ performance on habituation tasks predicts later cognitive development better than classical measures of infant development (e.g., McCall and Carriger 1993; but also see Klein‐Radukic and Zmyj 2023 and Robledo et al. 2010). In classical habituation paradigms, infants are repeatedly exposed to the same stimulus, and a decline in attention is taken as an index of learning. Infants who habituate more quickly—so‐called “fast habituators”—have been shown to perform better on later tests of intelligence, executive function, and academic achievement than “slow habituators” (Kavšek 2004).

Interestingly, the rate at which infants habituate appears to be unrelated to their curiosity. Poli, Ghilardi and colleagues (2024) showed that habituation performance is associated with processing speed, but not with infants’ sensitivity to information gain, suggesting that these measures tap into distinct cognitive processes. It also suggests that infants who may be slow at processing information may still demonstrate sensitivity to information gain. Whether such individual differences in infants’ sensitivity to information are predictive of later development remains an open question. Investigating this could provide further insight into early learning: it may not only be the speed at which infants process information that matters, but also what they choose to attend to.

In this longitudinal study (preregistered at OSF: https://doi.org/10.17605/OSF.IO/795ZW), we examined whether variations in infants’ curiosity, as reflected in their sensitivity to information, predict cognitive outcomes in childhood. The study was a follow‐up of the experiment by Poli and colleagues (2020) in which infants’ curiosity at 8 months was measured with the visual learning paradigm (Figure 1a–c). We used a hierarchical Bayesian model to assess infants’ sensitivity toward information by relating their looking time to the amount of information provided by each stimulus (Figure 1d,e). Upon reaching 3 years of age, children's intelligence was evaluated using a standardized intelligence test (WPPSI‐IV‐NL; Wechsler 2012; Figure 1f,g). The Wechsler Intelligence Scales (among which is the WPPSI‐IV‐NL, which is specifically designed for children aged 2.5 to 7 years) are among the most widely used instruments for assessing children's cognitive functioning (Wechsler 2012). The test provides estimates of global intellectual ability (Full Scale IQ) along with scores on three domains, including children's verbal comprehension, visual‐spatial reasoning, and working memory. IQ is a strong predictor of later academic achievement (Deary et al. 2007; Von Stumm et al. 2011). We additionally controlled for socioeconomic status (SES). We hypothesized that infants’ curiosity is related to childhood IQ, with infants who are more sensitive to the information showing higher intelligence 3 years later, thereby suggesting that infants’ curiosity is a key factor underlying positive cognitive outcomes.

## 2 Methods

This longitudinal study followed up on the infants that participated in the visual‐learning task by Poli and colleagues (2020). An additional 40 participants were collected with the same task for another study (Poli et al. 2023), adding up to 90 participants in total. Infants’ sensitivity to information was measured at 8 months of age for both of these studies. Here we obtained intelligence scores from the same participants when they were 3.5 years of age. The study outline is depicted in Figure 1.

The study was approved by the local ethics review board (ethical approval number: ECSW‐2020‐096). We deviated from the preregistered analyses in three ways: (1) instead of using the dichotomous estimate look‐away we employed a continuous estimate of looking time to determine infants’ sensitivity to information gain, (2) we applied an additive instead of a linear model in our main analysis, and (3) we introduced an outlier removal procedure. The rationale for each of these deviations is explained in detail in the sections below.

### 2.1 Participants

Eye‐tracking data were gathered from a sample of ninety 8‐month‐old infants (Poli et al. 2020, 2023). Of these infants, 20 did not provide usable data due to poor calibration or fussiness. At 3.5 years of age, intelligence scores were obtained from the same participants. Eight children dropped out for practical reasons such as relocation or time constraints, and two were excluded from the analyses due to incomplete administration of the intelligence test. The final sample consisted of 60 children, primarily White and middle‐class. The mean age (in months) of participants at the initial test was *M* = 7.9 (SD = 0.39) and *M* = 43.0 (SD = 0.36) at the follow‐up. The distribution of gender was equal (50% boys, 50% girls). In 93% of the cases, at least one of both caregivers completed a form of higher education (higher vocational or university education), and in 63% of the cases, both caregivers completed a form of higher education.

### 2.2 Measures

#### 2.2.1 Sensitivity to Information at 8 Months

Infants’ sensitivity to information was measured with a visual learning task during which looking behaviours were recorded using eye‐tracking (Figure 1a). The task was presented on a computer screen that was divided into four same‐sized quadrants (target locations) with a circle (cue location) in the middle. The task consisted of 16 sequences, each featuring one of eight unique stimuli (each stimulus was presented twice). Each sequence was composed of 15 trials, with each trial consisting of 4 phases: a cue phase (1000 ms) during which a stimulus was displayed in the cue location; an interstimulus phase (750 ms) during which the stimulus was not visible; a target phase (1500 ms) during which the stimulus was displayed in one of four target locations; and an intertrial interval (750 ms) during which the stimulus was not visible before it was presented again during the cue phase. All 15 trials were shown unless the infant looked away from the screen for longer than 1 s, triggering the sequence to stop. Once the infant looked back at the screen, the following sequence would start. Infants watched on average 7 sequences (SD = 2), and within each sequence, they watched 8 trials (SD = 4).

The location where the stimulus would reappear (see Figure 1b for an example sequence) during the target phase was statistically manipulated following three scenarios. In each scenario, the target location was predictable—it was possible for the infants to learn the most likely target location for each sequence—but predictability levels varied across scenarios (100%, 80%, or 60%). These different predictability levels were introduced to ensure variability in trial‐by‐trial information gain. In 4 out of 16 sequences, the stimulus always appeared at the same target location (100% predictable). In 6 out of 16 sequences, the stimulus appeared at the same target location for 80% of the trials while appearing randomly at one of the three other locations for the remaining 20%. In the remaining 6 out of 16 sequences, the stimulus appeared at the same target location for 60% of the trials while appearing randomly at one of the three other locations for the remaining 40%. Which quadrant served as the most likely target location was randomized across sequences and participants. Each quadrant served as the most likely target location in an equal number of sequences across the task (i.e., 25% each), and no two consecutive sequences shared the same most likely target location.

For each trial, we quantified the information gain it offered (Figure 1c) using Kullback–Leibler divergence (KL divergence). For each trial, KL divergence computes how much the new event (the stimulus appearing in the target location in the current trial) changes prior probabilities about the target location. For example, before the first trial, the stimulus is equally likely to appear in any four locations (probability is 25%). When in the first trial, the stimulus appears in the top left corner (location A in Figure 1a), the probability of this location increases (e.g., to 40%), while the probability of the other three locations decreases (e.g., to 20%). The more the new event changes these prior probabilities, the more information gain it offers. More information about these computations can be found in Poli and colleagues (2020).

Finally, to determine infants’ sensitivity to the information of each trial, we measured their looking time to the target stimulus on each trial. Then, by using a hierarchical Bayesian model (Figure 1d), we correlated the looking time towards each trial with the information gain of that specific trial. In this model, we controlled for stimulus surprise, sequence predictability, and trial number. This value was standardized, with higher values indicating higher sensitivity to information. Infants displayed individual differences in the extent to which their looking time to the target stimuli was correlated to the stimuli's information gain (Figure 1e).

As described in our preregistration, we initially planned to use the estimate of look‐away (a dichotomous measure indicating whether the infant kept looking until the end of the trial or looked away before the trial ended) in relation to information gain as our index of infants’ sensitivity to information. However, while look‐away is a good measure to estimate group effects, looking time turned out to be a more refined measure for studying individual differences (Poli, Ghilardi, et al. 2024). Thus, based on newly gained insights from the study by Poli, Ghilardi and colleagues (2024), we decided to use looking time during target presentation instead. We made this decision prior to the start of our analyses.

#### 2.2.2 IQ at 3.5 Years

We assessed children's IQ with the Wechsler Preschool and Primary Scale of Intelligence, Fourth Edition—Nederlandse bewerking (WPPSI‐IV‐NL; Hurks and Hendriksen 2020), the Dutch adaptation of the original WPPSI‐IV (Wechsler 2012). This standardized intelligence test is intended for children aged between 2.5 and 7 years and includes a version specifically designed for children up to 4 years. It comprises seven distinct tasks that provide insights into participants’ Full Scale Intelligence Quotient (Full Scale IQ) and three intelligence subindices, namely Verbal Comprehension Index (VCI), Visual Spatial Index (VSI), and Working Memory Index (WMI) (Figure 1f). In our primary analysis, we used the FSIQ, considered to be a representative indicator of global intellectual functioning, as our main outcome measure (Wechsler 2012).

#### 2.2.3 SES

SES was determined by the average education level of the caregiver(s). For both caregivers (if applicable, *n* = 1 child was raised by a single caregiver), we collected information on their highest level of education, ranging from 1 (secondary education), 2 (intermediate vocational education), 3 (higher vocational education) to 4 (university education). SES was determined by the average education level of the caregiver(s).

### 2.3 Procedure

Families were invited to the Baby & Child Research Center in Nijmegen, an urban city in the Netherlands, twice—once when their child was 8 months old and once when their child was 3.5 years old. At the first visit, participants’ eye movements were measured during the visual learning task through which infants’ sensitivity to information was assessed. During the task, infants sat in a baby seat that was positioned on the caregiver's lap in front of the eye‐tracker (Tobii X300) monitor that displayed the stimuli. Caregivers were instructed to refrain from interacting with their child and, when their infant sought their attention, not to attempt to redirect their attention back to the screen. The experiment ended when the infant had watched all 16 sequences or when they became fussy.

At the second visit, the WPPSI‐IV‐NL was administered to assess children's intelligence. During the test, children sat behind a table across from an experimenter. The caregiver was positioned at a table behind their child, ensuring proximity while minimizing their influence on the child's behaviour. While the researcher administered the intelligence test, the caregiver completed a set of questionnaires on a laptop. The intelligence test lasted approximately 45 to 60 min. Approximately halfway through the session, typically after completing task five or earlier if children displayed signs of fatigue, a 15‐min break was allocated. Additional information was collected, including a computer‐based game that was played by the child after the intelligence test and a questionnaire that was filled in by the caregiver while the intelligence test was administered. The results obtained from these measures were part of a different study and fall outside the scope of the present paper.

At both visits, there was some time reserved prior to the experiment to familiarize the child with the experimenter. After the experiment, the caregiver was debriefed, and the child received a gift. At the first visit, the caregiver could choose between a monetary reward of 10 euros or a children's book. At the second visit, the gift consisted of a ‘diploma’ and either 20 euros or two children's books.

### 2.4 Statistical Analysis

All analyses were performed in R (Version 4.3.1). The data and analysis scripts are available on the Radboud Data Repository at https://doi.org/10.34973/31ee‐x555.

Prior to the analyses, we performed an outlier removal procedure for individuals who scored below or over 3 standard deviations from the mean on the sensitivity to information measure. This outlier removal procedure was set before the analysis but was not specified in the preregistration. We decided on the threshold of 3 standard deviations because it carries no negative consequence for type I error (Bakker and Wicherts 2014). It led to the exclusion of one infant.

#### 2.4.1 Group‐Level Analysis Relating Information Gain to Looking Time

Before running the main analysis on individual differences, we performed the group‐level analyses from Poli and colleagues (2020) and related surprise, predictability, and information gain of a trial to infants’ looking time. For this, we used a linear mixed model with looking time as the dependent variable and surprise, predictability, and information gain as the independent variables, controlling for saccadic latency, time (trial number) and the random intercept of looking time (to account for individual differences in looking time). While surprise measures the (un)expectedness of a single event (e.g., the target appearing at location A on trial 5), predictability tracks the expectedness of the overall sequence up to that trial. Information gain is different from surprise and predictability because it captures how much new knowledge can be extracted from a trial, rather than how unexpected it is (surprise) or how regular the environment has been so far (predictability). A detailed analysis of these metrics is beyond the scope of this paper, but more information can be found in Poli and colleagues (2020).

Additionally, to assess whether infants learnt to predict the most likely target location, we compared saccadic latencies of predictable and unpredictable events using a linear mixed model. Saccadic latency was entered as the dependent variable, with trial type (predictable vs. unpredictable) as the independent variable. In this dichotomous measure of predictability, a trial was considered to be predictable when the target appears in the high‐probability location and unpredictable when it appears in any of the low‐probability locations. We controlled for trial number and also included the random intercept of the participant in the model to account for individual differences in saccadic latencies. In the event that infants learn the statistical regularities, we would expect infants to direct their attention faster to predictable compared to unpredictable trials.

#### 2.4.2 Main Analysis Relating Individual Differences in Sensitivity to Information Gain to IQ

We examined the relation between infants’ sensitivity to information and childhood intelligence while controlling for SES using an additive model. In this model, participants’ Full Scale IQ was included as the dependent variable, infants’ sensitivity to information as the independent variable, and SES (indexed by the average educational level of caregiver[s]) was added as a covariate. SES was included in the model because previous studies demonstrated links between SES and IQ (with children growing up in higher SES households showing higher IQ scores; Bradley and Corwyn 2002; Von Stumm and Plomin 2015), as well as between SES and curiosity (Shah et al. 2018, 2023).

Although we preregistered the use of a linear model, based on additional statistical insights gained during previous studies (Poli, Ghilardi, et al. 2024) and prior to data analysis, we decided to use this additive model instead. The additive model has the advantage of being more flexible while keeping the desired properties of a linear model. It provides a clearer and more accurate representation of the underlying trends and patterns in the data, thus allowing for the detection of linear effects as well as non‐linear ones. For transparency, however, we also fitted the data to our preregistered linear model. This model yielded no evidence for a linear relation between infants’ sensitivity to information and childhood intelligence (*β* = 2.01, *t* = 1.12, *p* = 0.27). Consistently, the additive model showed a better fit (AIC = 459.73) than the preregistered linear model (AIC = 471.97).

Additionally, as additive models have a risk of overfitting, we explored the pattern that resulted from the additive model further by (1) fitting the data to a more constrained exponential model and (2) conducting a stratified analysis in which we observed the relationships (using additive models with the same specifications as in the main analysis) between infants’ sensitivity to information gain and later IQ separately for three groups of infants with different levels of sensitivity to information.

#### 2.4.3 Exploratory Analysis Relating Individual Differences in Sensitivity to Information to IQ Subindices

Lastly, for exploratory purposes, we examined the relation between infants’ sensitivity to information and the intelligence subindices VCI, VSI, and WMI. For this, we ran three separate additive models including either VCI, VSI or WMI as the dependent variable, infants’ sensitivity to information as the independent variable, and SES as the covariate.

## 3 Results

### 3.1 Descriptives and Results of the Group‐Level Analysis

At the group level, information gain significantly predicted looking times (*β* = 66.21, SE = 20.84, *t*(2569) = 3.18, *p* = 0.002); the greater the information gain a trial offered, the longer infants looked at it. Surprise did not significantly predict looking times (*β* = −17.94, SE = 20.21, *t*(2567) = −0.89, *p* = 0.37). These findings are in line with Poli and colleagues (2020). However, unlike the findings reported by Poli and colleagues (2020), predictability also significantly predicted infants’ looking time (*β* = −57.76, SE = 19.88, *t*(2593) = −2.91, *p* = 0.004); when the predictability was lower, infants looked longer. Regarding saccadic latency in relation to the dichotomous measure of predictability, we found that infants were significantly faster to orient to predictable trials compared to unpredictable ones (*β* = 180.26, SE = 24.26, *t* = −7.43, *p* < 0.001): Infants were 180 ms faster at looking at predictable compared to unpredictable trials, suggesting that infants learnt the most likely target location.

The average IQ score of children at age 3.5 years was *M* = 106 (SD = 12.6). The mean scores on the IQ subindices were as follows: *M* = 106 (SD = 15.6) for the VCI, *M* = 103 (SD = 11.3) for the VSI and *M* = 109 (SD = 9.5) for the WMI. VCI showed a significant positive correlation with VSI (*r* = 0.38, *p* = 0.004) and WMI (*r* = 0.53, *p* < 0.001). The correlation between VSI and WMI was not statistically significant (*r* = 0.23, *p* = 0.087).

### 3.2 Results of the Main Analysis

At the individual level, infants differed in the degree to which their looking time correlated with the information gain that was provided by that stimulus (Figure 1e). To link these individual differences in infants’ curiosity to their later cognitive capabilities, we used a generalized additive model relating measures of sensitivity to information with childhood IQ while controlling for differences in SES between the infants’ families. By applying this additive model, we deviated from the preregistration that describes using a linear model. The details concerning this change in statistical approach are discussed in the Methods section (Statistical Analysis).

**FIGURE 2:**
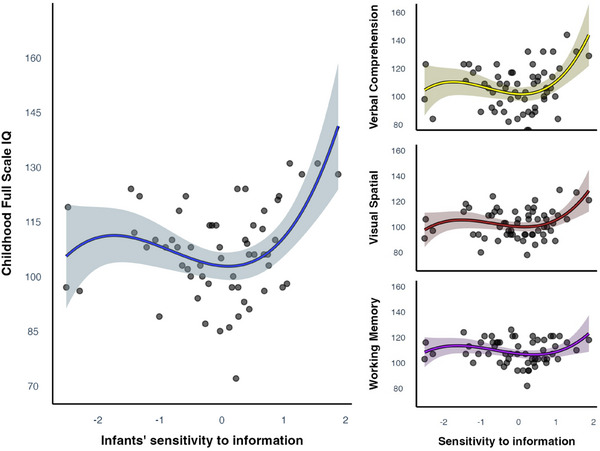
Results of the generalized additive model relating infants’ sensitivity to information with childhood Full Scale IQ and intelligence subindices. *Note*: Infants’ sensitivity to information was significantly related to the Full Scale IQ scores in childhood. The relation between infants’ sensitivity to information and the IQ subindices followed a similar non‐linear relation. Of those, only the relation between infants’ sensitivity to information and the Verbal Comprehension Index was significant. The shaded areas surrounding the plots represent confidence intervals.

Individual differences in infants’ sensitivity to information significantly predicted childhood IQ (*F* = 3.87, edf (effective degrees of freedom) = 3.58, *p* = 0.006, see Figure 2), with the model explaining 22% of the variance in childhood intelligence (adjusted R^2^). SES did not have a significant effect on IQ (*t* = 0.98, *p* = 0.33). These results were not affected when controlling for other attention measures such as infants’ sustained attention, learning performance, or processing speed (see Supplementary Analyses S1). To assess the unique contribution of infants' sensitivity to information, we computed the partial adjusted *R* ^2^ using *gam.hp* (Lai et al. 2024). This analysis showed that infants’ sensitivity to information uniquely accounted for 22% of the variance in childhood IQ (the unique variance explained by SES was 0.18%).

Several aspects of the findings suggest that the relationship between infants’ sensitivity to information gain and intelligence is non‐linear: First, the linear model that we fitted to the data revealed no significant relationship (*β* = 2.01, *t* = 1.12, *p* = 0.27). Second, the number of effective degrees of freedom was significantly different from 1, indicating that the relationship is not adequately described by a linear slope (Hastie and Tibshirani 1986). Finally, visual inspection of the data pattern suggests that the relationship was primarily present among children with higher curiosity scores, whereas little to no association was observed at lower curiosity levels.

To examine this pattern further, and to account for the fact that additive models have a risk of overfitting, we examined a more constrained exponential model. The results of this alternative model demonstrated that the exponential relationship between curiosity and IQ was significant (estimate = 3.75, *t* = 2.74, *p* = 0.008), but that it explained less variance in childhood intelligence compared to the additive model (adjusted *R* ^2^ = 10%). In addition to the exponential model, and to further confirm the non‐linear relationship, we conducted stratified analyses by dividing the sample into three equal groups based on infants’ degree of sensitivity to information. One third of the infants with the lowest sensitivity to information scores were placed in the *Low* curiosity group, the middle third in the *Medium* curiosity group and the highest third in the *High* curiosity group. The results supported the pattern observed in the main model. In the High curiosity group, sensitivity to information significantly predicted IQ (*F* = 10.26, *p* = 0.005, *n* = 20), with the model explaining 34% of the variance (adjusted *R* ^2^). In contrast, the effect of sensitivity to information was not significant in the Medium (*F* = 0.03, *p* = 0.86; adj. *R* ^2^ = −8.5%, *n* = 19) or Low (*F* = 0.53, *p* = 0.58; adj. *R* ^2^ = −0.41%, *n* = 20) curiosity groups.

### 3.3 Results of the Exploratory Analysis

We also explored the relation between infants’ sensitivity to information and each intelligence subindex separately. There was a significant non‐linear relation between infants’ sensitivity to information and their later Verbal Comprehension Index (VCI; *M* = 106, SD = 15.6, *F* = 2.96, edf = 3.23, *p* = 0.028). The relations between infants’ sensitivity to information and the Visual Spatial Index (VSI; *M* = 103, SD = 11.3) and Working Memory Index (WMI; *M* = 109, SD = 9.5) followed a similar non‐linear pattern but were not significant (*F* = 2.05, edf = 2.88, *p* = 0.108 and *F* = 1.2, edf = 2.97, *p* = 0.286).

## 4 Discussion

Research on infant curiosity and its role in learning and early development has received increasing attention in recent years (e.g., Chu and Schulz 2020; Hunnius and Poli forthcoming; Jirout et al. 2024; Kidd and Hayden 2015; Perez and Feigenson 2021). Previous studies have shown that children are curious from a very early age, and that infants differ in the degree to which they are curious (Addyman and Mareschal 2013; Bazhydai et al. 2020; Kidd et al. 2012; Poli, Ghilardi, et al. 2024; Poli et al. 2020; Ruggeri et al. 2019; Twomey and Westermann 2018). In this study, we examined the longitudinal link between individual differences in early manifestations of curiosity, as reflected in infants’ sensitivity to information gain, and subsequent cognitive development. We tested 8‐month‐old infants on a visual learning task and extracted individual differences in their sensitivity to information using a hierarchical Bayesian model. Three years later, we tested the same children on their intelligence. We showed that infants’ sensitivity to information relates to childhood cognitive abilities over a period of almost 3 years. As such, these findings provide initial empirical evidence that supports the longitudinal implications suggested by the learning progress hypothesis, a theoretical framework of curiosity (Oudeyer et al. 2016; Ten et al. 2021).

More specifically, the non‐linear relationship that we found between curiosity and IQ suggests that children who displayed the greatest curiosity as infants tend to have a more favourable cognitive development, while infants displaying lower levels of curiosity are not necessarily at a disadvantage. This interpretation is supported by additional analyses that demonstrated that the relationship between sensitivity to information gain and IQ holds primarily for the group of children who exhibited high curiosity as infants. Curiosity might thus act as a boost factor—enriching further development—but not as a risk factor; that is, a lack of curiosity does not necessarily lead to lower cognitive capacity. It is important to consider, however, that our sample contained relatively many children with above‐average IQ scores: only 10% of our participants had intelligence scores that were below average, and those were only minimally below the cut‐off (the clinical cutoff for below‐average IQ scores on the WPPSI‐IV‐NL is typically set at 90; Ruiter et al. 2017; Wechsler 2012). Moreover, our sample consisted predominantly of children growing up in high SES households (Bradley and Corwyn 2002; Von Stumm and Plomin 2015). As a result, our findings may be capturing primarily the ‘boost effect’ of curiosity among children who are performing well cognitively, while possible associations at the lower end of the distribution may not be visible. Future studies with more socioeconomically and cognitively diverse samples of children will help to clarify the relation between an infant's curiosity and later IQ.

Our findings imply a potentially cascading effect, where infants who pay more attention to stimuli they can learn from may be exposed to more learning opportunities, which in turn enhances their cognitive development over time (Bornstein et al. 2013; Fry and Hale 1996; Iverson 2022; Masten and Cicchetti 2010). Consistent with this interpretation, we found that the effect of infants’ curiosity on their later IQ was primarily driven by the Verbal Comprehension Index, rather than by the subindices for visual‐spatial reasoning or working memory. Whereas the assessment of verbal comprehension mainly relies on accumulated, explicitly taught knowledge (e.g., vocabulary, general world knowledge) that must be acquired through direct exposure, the visual‐spatial and working memory domains—assessed through solving puzzles, recreating geometric patterns, or recalling stimuli—represent more fundamental, domain‐general cognitive abilities that may be less dependent on specific learning input. Children who display more curiosity towards their surroundings at a young age may prompt more relevant language input—for instance, through the contingent verbal responses of their caregivers (Jirout et al. 2024; Tamis‐LeMonda et al. 2013)—which in turn scaffolds their understanding and acquisition of words and facts (Kartushina et al. 2022; Masek et al. 2021). A recent study provides support for the existence of such an effect in children's verbal development. Van der Klis and colleagues (2024) examined the dyadic interaction between infants and caregivers during 6 min of free‐play and found that infants’ pointing behaviour that was followed by a verbal response from their caregiver predicted later vocabulary outcomes. In contrast, infants' gestures that did not elicit such a verbal response were negatively related to later vocabulary scores. Thus, through their tendency to seek information, infants may create their own learning opportunities and thereby stimulate their cognitive growth.

An important direction for future research is to better understand how curiosity may influence specific cognitive subdomains. The current analyses of the intelligence subindices were exploratory and not guided by specific hypotheses. Therefore, the outcomes should be interpreted with caution. Specifically, while we found a significant association between infants’ curiosity and later verbal comprehension (Mani and Ackermann 2018), this does not imply that the lack of significant associations with the other subindices should be interpreted as evidence for the absence of such effects. In fact, the relationships that we observed between curiosity and the three cognitive subdomains followed similar patterns. The non‐significant relationships between infants’ curiosity and their later working memory and visual‐spatial reasoning could potentially be due to the lower variability levels that were observed on these scales in our sample. Additionally, as the subindices are correlated components of a composite Full Scale IQ score (which was our primary outcome), we caution against overinterpreting the significance of one subindex over others. Nonetheless, the current pattern of findings may help refine future hypotheses about which cognitive domains are most strongly impacted by differences in curiosity. Relatedly, future research should aim to identify the sources of the individual differences in early curiosity. This includes investigating the degree to which infants are born with a tendency to focus on informative events, what the neural basis for this might be, and whether environmental factors, such as parental responsiveness to infants’ information‐seeking behaviours, influence the development of curiosity.

Until recently, individual differences in fundamental cognitive mechanisms early in life were difficult to quantify (Piantadosi et al. 2014), and variability was therefore often discarded. Poli and colleagues (Poli, Ghilardi, et al. 2024; Poli et al. 2020) introduced a hierarchical Bayesian model to harness these variations in early behavioural measures to extract meaningful differences in cognitive processes that are more precise than what could be perceived directly from the behavioural data alone. Here, we adopted this novel model‐based approach to obtain individual differences in infants’ sensitivity to information—as inferred from their looking behaviours in a visual learning task—to examine their relationship with later cognitive outcomes. We show that this approach can be applied to predict real‐world developmental outcomes from individual differences in processing abilities in infancy using a longitudinal design.

In a nutshell, how do our findings further our understanding of cognitive development and the role of infants’ processing abilities therein? Prior research demonstrated that infants as young as 8 months selectively allocate their attention towards stimuli from which they can gain information and that infants display individual differences in this mechanism (Poli, Ghilardi, et al. 2024; Poli et al. 2020). Building on this, we show that early‐existing individual differences in curiosity‐driven learning play an important role in cognitive development and allow predicting differences in cognitive capacity over a time span of almost 3 years, supporting the direction modern theories are taking in emphasizing the role of infant curiosity in early learning. Benefiting from this discovery, these results suggest that finding ways to stimulate curiosity might be a promising avenue for boosting exploratory behaviour and supporting learning in early childhood.

## Author Contributions

**Eline R. de Boer**: conceptualization, formal analysis, investigation, methodology, project administration, writing – original draft. **Francesco Poli**: conceptualization, formal analysis, methodology, writing – review and editing. **Marlene Meyer**: conceptualization, methodology, supervision, writing – review and editing. **Rogier B. Mars**: conceptualization. **Sabine Hunnius**: conceptualization, methodology, supervision, writing – review and editing.

## Funding

This work was supported by an NWO VICI grant to Sabine Hunnius (grant number VI.C.191.022).

## Ethics Statement

The study was approved by the local ethics review board (Ethical approval number: ECSW‐2020‐096). Authors give permission to reproduce material from other sources.

## Conflicts of Interest

The authors declare no conflicts of interest.

## Supporting information




**Supporting File 1**: desc70090‐sup‐0001‐SuppMat.docx

## Data Availability

All data are available in the main text or the supplementary materials. The data are shared for scientific use only and therefore only accessible for registered users. Data and analytic code are available at the following URL: https://doi.org/10.34973/31ee‐x555.

## References

[desc70090-bib-0001] Addyman, C. , and D. Mareschal . 2013. “Local Redundancy Governs Infants' Spontaneous Orienting to Visual‐Temporal Sequences.” Child Development 84, no. 4: 1137–1144. 10.1111/cdev.12060. 23432603

[desc70090-bib-0002] Andersen, M. M. , J. Kiverstein , M. Miller , and A. Roepstorff . 2022. “Play in Predictive Minds: A Cognitive Theory of Play.” Psychological Review 130, no. 2: 462–479. 10.1037/rev0000369. 35708932

[desc70090-bib-0003] Baer, C. , and C. Kidd . 2022. “Learning With Certainty in Childhood.” Trends in Cognitive Sciences 26, no. 10: 887–896. 10.1016/j.tics.2022.07.010. 36085134

[desc70090-bib-0004] Baillargeon, R. , E. S. Spelke , and S. Wasserman . 1985. “Object Permanence in Five‐Month‐Old Infants.” Cognition 20, no. 3: 191–208. 10.1016/0010-0277(85)90008-3. 4064606

[desc70090-bib-0005] Bakker, M. , and J. M. Wicherts . 2014. “Outlier Removal, Sum Scores, and the Inflation of the Type I Error Rate in Independent Samples T Tests: The Power of Alternatives and Recommendations.” Psychological Methods 19, no. 3: 409–427. 10.1037/met0000014. 24773354

[desc70090-bib-0006] Bazhydai, M. , K. Twomey , and G. Westermann . 2019. “Curiosity and Exploration.” *In* Encyclopedia of Infant and Early Childhood Development, edited by M. M. Haith and J. B. Benson . 2nd ed. Elsevier. https://research.manchester.ac.uk/en/publications/curiosity‐and‐exploration.

[desc70090-bib-0007] Bazhydai, M. , G. Westermann , and E. Parise . 2020. “'I Don't Know but I Know Who to Ask': 12‐Month‐Olds Actively Seek Information From Knowledgeable Adults.” Developmental Science 23, no. 5: e12938. 10.1111/desc.12938. 31954092

[desc70090-bib-0008] Berlyne, D. E. 1966. “Curiosity and Exploration: Animals Spend Much of Their Time Seeking Stimuli Whose Significance Raises Problems for Psychology.” Science 153, no. 3731: 25–33. 10.1126/science.153.3731.25. 5328120

[desc70090-bib-0009] Bornstein, M. H. , C. S. Hahn , and D. Wolke . 2013. “Systems and Cascades in Cognitive Development and Academic Achievement.” Child Development 84, no. 1: 154–162. 10.1111/j.1467-8624.2012.01849.x. 22974268 PMC3525805

[desc70090-bib-0010] Bornstein, M. H. , and M. D. Sigman . 1986. “Continuity in Mental Development From Infancy.” Child Development 57, no. 2: 251–274. 10.2307/1130581. 3956312

[desc70090-bib-0011] Bradley, R. H. , and R. F. Corwyn . 2002. “Socioeconomic Status and Child Development.” Annual Review of Psychology 53, no. 1: 371–399. 10.1146/annurev.psych.53.100901.135233. 11752490

[desc70090-bib-0012] Chen, X. , K. E. Twomey , and G. Westermann . 2022. “Curiosity Enhances Incidental Object Encoding in 8‐Month‐Old Infants.” Journal of Experimental Child Psychology 223: 105508. 10.1016/j.jecp.2022.105508. 35850003

[desc70090-bib-0013] Chu, J. , and L. E. Schulz . 2020. “Play, Curiosity, and Cognition.” Annual Review of Developmental Psychology 2, no. 1: 317–343. 10.1146/annurev-devpsych-070120-014806.

[desc70090-bib-0014] Cuevas, K. , and M. A. Bell . 2014. “Infant Attention and Early Childhood Executive Function.” Child Development 85, no. 2: 397–404. 10.1111/cdev.12126. 23711103 PMC3766399

[desc70090-bib-0015] Deary, I. J. , S. Strand , P. Smith , and C. Fernandes . 2007. “Intelligence and Educational Achievement.” Intelligence 35, no. 1: 13–21. 10.1016/j.intell.2006.02.001.

[desc70090-bib-0016] Dougherty, T. M. , and M. M. Haith . 1997. “Infant Expectations and Reaction Time as Predictors of Childhood Speed of Processing and IQ.” Developmental Psychology 33, no. 1: 146–155. 10.1037/0012-1649.33.1.146. 9050399

[desc70090-bib-0017] Dubey, R. , and T. L. Griffiths . 2020. “Reconciling Novelty and Complexity Through a Rational Analysis of Curiosity.” Psychological Review 127, no. 3: 455–476. 10.1037/rev0000175. 31868394

[desc70090-bib-0018] Fantz, R. L. 1964. “Visual Experience in Infants: Decreased Attention to Familiar Patterns Relative to Novel Ones.” Science 146, no. 3644: 668–670. 10.1126/science.146.3644.668. 14191712

[desc70090-bib-0019] Fry, A. F. , and S. Hale . 1996. “Processing Speed, Working Memory, and Fluid Intelligence: Evidence for a Developmental Cascade.” Psychological Science 7, no. 4: 237–241. 10.1111/j.1467-9280.1996.tb00366.x.

[desc70090-bib-0020] Gottlieb, J. , P.‐Y. Oudeyer , M. Lopes , and A. Baranes . 2013. “Information‐Seeking, Curiosity, and Attention: Computational and Neural Mechanisms.” Trends in Cognitive Sciences 17, no. 11: 585–593. 10.1016/j.tics.2013.09.001. 24126129 PMC4193662

[desc70090-bib-0021] Hastie, T. , and R. Tibshirani . 1986. “Generalized Additive Models.” Statistical Science 1, no. 3: 297–310. 10.1177/096228029500400302 8548102

[desc70090-bib-0022] Hitzert, M. M. , K. N. J. A. Van Braeckel , A. F. Bos , S. Hunnius , and R. H. Geuze . 2014. “Early Visual Attention in Preterm and Fullterm Infants in Relation to Cognitive and Motor Outcomes at School Age: An Exploratory Study.” Frontiers in Pediatrics 2: 106. 10.3389/fped.2014.00106. 25340045 PMC4186265

[desc70090-bib-0023] Hunnius, S. , and F. Poli . Forthcoming. “Curious Minds: Learning and Exploration in Early development.” In OUP Handbook of Perceptual Development, edited by S. Johnson . Oxford University Press.

[desc70090-bib-0024] Hurks, P. , and J. Hendriksen . 2020. Technische Handleiding Wechsler Preschool and Primary Scale of Intelligence—Fourth Edition—Nederlandstalige Bewerking (WPPSI‐IV NL). Pearson.

[desc70090-bib-0025] Iverson, J. M. 2022. “Developing Language in a Developing Body, Revisited: The Cascading Effects of Motor Development on the Acquisition of Language.” WIREs Cognitive Science 13, no. 6: e1626. 10.1002/wcs.1626. 36165333 PMC12333486

[desc70090-bib-0026] Jirout, J. J. , N. S. Evans , and L. K. Son . 2024. “Curiosity in Children Across Ages and Contexts.” Nature Reviews Psychology 3: 622–635. 10.1038/s44159-024-00346-5.

[desc70090-bib-0027] Kartushina, N. , N. Mani , A. Aktan‐Erciyes , et al. 2022. “COVID‐19 First Lockdown as a Window Into Language Acquisition: Associations Between Caregiver‐Child Activities and Vocabulary Gains.” Language Development Research 2, no. 1: 1–36. 10.31234/osf.io/5ejwu.

[desc70090-bib-0028] Kavšek, M. 2004. “Predicting Later IQ From Infant Visual Habituation and Dishabituation: A Meta‐Analysis.” Journal of Applied Developmental Psychology 25, no. 3: 369–393. 10.1016/j.appdev.2004.04.006.

[desc70090-bib-0029] Kidd, C. , and B. Y. Hayden . 2015. “The Psychology and Neuroscience of Curiosity.” Neuron 88, no. 3: 449–460. 10.1016/j.neuron.2015.09.010. 26539887 PMC4635443

[desc70090-bib-0030] Kidd, C. , S. T. Piantadosi , and R. N. Aslin . 2012. “The Goldilocks Effect: Human Infants Allocate Attention to Visual Sequences That Are Neither Too Simple Nor Too Complex.” PLoS ONE 7, no. 5: e36399. 10.1371/journal.pone.0036399. 22649492 PMC3359326

[desc70090-bib-0031] Klein‐Radukic, S. , and N. Zmyj . 2023. “The Predictive Value of the Cognitive Scale of the Bayley Scales of Infant and Toddler Development‐III.” Cognitive Development 65: 101291. 10.1016/j.cogdev.2022.101291.

[desc70090-bib-0032] Lai, J. , J. Tang , T. Li , A. Zhang , and L. Mao . 2024. “Evaluating the Relative Importance of Predictors in Generalized Additive Models Using the Gam.Hp R Package.” Plant Diversity 46, no. 4: 542–546. 10.1016/j.pld.2024.06.002. 39280972 PMC11390626

[desc70090-bib-0033] Mani, N. , and L. Ackermann . 2018. “Why Do Children Learn the Words They Do?” Child Development Perspectives 12, no. 4: 253–257. 10.1111/cdep.12295.

[desc70090-bib-0034] Masek, L. R. , B. T. M. McMillan , S. J. Paterson , C. S. Tamis‐LeMonda , R. M. Golinkoff , and K. Hirsh‐Pasek . 2021. “Where Language Meets Attention: How Contingent Interactions Promote Learning.” Developmental Review 60: 100961. 10.1016/j.dr.2021.100961.

[desc70090-bib-0035] Masten, A. S. , and D. Cicchetti . 2010. “Developmental Cascades.” Development and Psychopathology 22, no. 3: 491–495. 10.1017/S0954579410000222. 20576173

[desc70090-bib-0036] McCall, R. B. , and M. S. Carriger . 1993. “A Meta‐Analysis of Infant Habituation and Recognition Memory Performance as Predictors of Later IQ.” Child Development 64, no. 1: 57–79. 10.2307/1131437. 8436038

[desc70090-bib-0037] Muentener, P. , E. Herrig , and L. Schulz . 2018. “The Efficiency of Infants' Exploratory Play Is Related to Longer‐Term Cognitive Development.” Frontiers in Psychology 9: 635. 10.3389/fpsyg.2018.00635. 29904360 PMC5991261

[desc70090-bib-0038] Oudeyer, P.‐Y. 2018. "Computational Theories of Curiosity‐Driven Learning." Preprint, arXiv, February 28. 10.48550/ARXIV.1802.10546.

[desc70090-bib-0039] Oudeyer, P.‐Y. , J. Gottlieb , and M. Lopes . 2016. “Intrinsic Motivation, Curiosity, and Learning: Theory and Applications in Educational Technologies.” In Progress in Brain Research, edited by B. Studer and S. Knecht , 257–284. Elsevier. 10.1016/bs.pbr.2016.05.005. 27926442

[desc70090-bib-0040] Oudeyer, P.‐Y. , F. Kaplan , and V. V. Hafner . 2007. “Intrinsic Motivation Systems for Autonomous Mental Development.” IEEE Transactions on Evolutionary Computation 11, no. 2: 265–286. 10.1109/TEVC.2006.890271.

[desc70090-bib-0041] Perez, J. , and L. Feigenson . 2021. “Stable Individual Differences in Infants' Responses to Violations of Intuitive Physics.” Proceedings of the National Academy of Sciences 118, no. 27: e2103805118. 10.1073/pnas.2103805118. PMC8271639 34183399

[desc70090-bib-0042] Piaget, J. 1952. The Origins of Intelligence in Children. W. W. Norton & Company, Inc.

[desc70090-bib-0043] Piantadosi, S. T. , C. Kidd , and R. Aslin . 2014. “Rich Analysis and Rational Models: Inferring Individual Behavior From Infant Looking Data.” Developmental Science 17, no. 3: 321–337. 10.1111/desc.12083. 24750256 PMC3996510

[desc70090-bib-0044] Poli, F. , T. Ghilardi , R. Beijers , et al. 2024. “Individual Differences in Processing Speed and Curiosity Explain Infant Habituation and Dishabituation Performance.” Developmental Science 27: e13460. 10.1111/desc.13460. 38155558

[desc70090-bib-0045] Poli, F. , T. Ghilardi , R. B. Mars , M. Hinne , and S. Hunnius . 2023. “Eight‐month‐Old Infants Meta‐Learn by Downweighting Irrelevant Evidence.” Open Mind 7: 141–155. 10.1162/opmi_a_00079. 37416070 PMC10320826

[desc70090-bib-0046] Poli, F. , J. X. O'Reilly , R. B. Mars , and S. Hunnius . 2024. “Curiosity and the Dynamics of Optimal Exploration.” Trends in Cognitive Sciences 28: 441–453. 10.1016/j.tics.2024.02.001. 38413257

[desc70090-bib-0047] Poli, F. , G. Serino , R. B. Mars , and S. Hunnius . 2020. “Infants Tailor Their Attention to Maximize Learning.” Science Advances 6, no. 39: eabb5053. 10.1126/sciadv.abb5053. 32967830 PMC7531891

[desc70090-bib-0048] Robledo, M. , G. Deák , and T. Kolling . 2010. “Infants' Visual Processing of Faces and Objects: Age‐Related Changes in Interest, and Stability of Individual Differences.” Proceedings of the Annual Meeting of the Cognitive Science Society 32, no. 32: 2482–2487.

[desc70090-bib-0049] Rose, S. A. , and J. F. Feldman . 1997. “Memory and Speed: Their Role in the Relation of Infant Information Processing to Later IQ.” Child Development 68, no. 4: 630–641. 10.2307/1132115. 9306643

[desc70090-bib-0050] Rose, S. A. , and I. F. Wallace . 1985. “Visual Recognition Memory: A Predictor of Later Cognitive Functioning in Preterms.” Child Development 56, no. 4: 843–852. 10.2307/1130096. 4042748

[desc70090-bib-0051] Ruggeri, A. , N. Swaboda , Z. L. Sim , and A. Gopnik . 2019. “Shake It Baby, but Only When Needed: Preschoolers Adapt Their Exploratory Strategies to the Information Structure of the Task.” Cognition 193: 104013. 10.1016/j.cognition.2019.104013. 31280062

[desc70090-bib-0052] Ruiter, S. A. J. , P. P. M. Hurks , and M. E. Timmerman . 2017. “Iq‐Score Is Dringend Aan Modernisering Toe.” Kind & Adolescent Praktijk 16, no. 1: 16–23. 10.1007/s12454-017-0005-y.

[desc70090-bib-0053] Shah, P. E. , K. Hirsh‐Pasek , M. Spinelli , et al. 2023. “Ecological Contexts Associated With Early Childhood Curiosity: Neighborhood Safety, Home and Parenting Quality, and Socioeconomic Status.” Frontiers in Psychology 14: 986221. 10.3389/fpsyg.2023.986221. 36925599 PMC10011070

[desc70090-bib-0054] Shah, P. E. , H. M. Weeks , B. Richards , and N. Kaciroti . 2018. “Early Childhood Curiosity and Kindergarten Reading and Math Academic Achievement.” Pediatric Research 84, no. 3: 380–386. 10.1038/s41390-018-0039-3. 29884846 PMC6203666

[desc70090-bib-0055] Stahl, A. E. , and L. Feigenson . 2015. “Observing the Unexpected Enhances Infants' Learning and Exploration.” Science 348, no. 6230: 91–94. 10.1126/science.aaa3799. 25838378 PMC5861377

[desc70090-bib-0056] Tamis‐LeMonda, C. S. , Y. Kuchirko , and L. Tafuro . 2013. “From Action to Interaction: Infant Object Exploration and Mothers' Contingent Responsiveness.” IEEE Transactions on Autonomous Mental Development 5, no. 3: 202–209. 10.1109/TAMD.2013.2269905.

[desc70090-bib-0057] Ten, A. , P. Kaushik , P.‐Y. Oudeyer , and J. Gottlieb . 2021. “Humans Monitor Learning Progress in Curiosity‐Driven Exploration.” Nature Communications 12, no. 1: 5972. 10.1038/s41467-021-26196-w. PMC8514490 34645800

[desc70090-bib-0058] Twomey, K. E. , and G. Westermann . 2018. “Curiosity‐Based Learning in Infants: A Neurocomputational Approach.” Developmental Science 21, no. 4: e12629. 10.1111/desc.12629. 29071759 PMC6032944

[desc70090-bib-0059] Van der Klis, A. , C. Junge , F. Adriaans , and R. Kager . 2024. “The Role of Dyadic Combinations of Infants' Behaviors and Caregivers' Verbal and Multimodal Responses in Predicting Vocabulary Outcomes.” Infancy 30, no. 1: e12626. 10.1111/infa.12626. 39322979 PMC11649878

[desc70090-bib-0060] Von Stumm, S. , B. Hell , and T. Chamorro‐Premuzic . 2011. “The Hungry Mind: Intellectual Curiosity Is the Third Pillar of Academic Performance.” Perspectives on Psychological Science 6, no. 6: 574–588. 10.1177/1745691611421204. 26168378

[desc70090-bib-0061] Von Stumm, S. , and R. Plomin . 2015. “Socioeconomic Status and the Growth of Intelligence From Infancy Through Adolescence.” Intelligence 48: 30–36. 10.1016/j.intell.2014.10.002. 26640306 PMC4641149

[desc70090-bib-0062] Vygotsky, L. S. , and M. Cole . 1978. Mind in Society: Development of Higher Psychological Processes. Harvard University Press.

[desc70090-bib-0063] Wechsler, D. 2012. Technical and Interpretative Manual Wechsler Preschool and Primary Scale of Intelligence—Fourth Edition (WPPSI‐IV). Pearson Inc.

