# Supplementary material for: Individual Differences in Infants’ Curiosity Are Linked to Cognitive Capacity in Early Childhood
Source: Dev Sci. 2025 Nov 7;29(1):e70090. doi: 10.1111/desc.70090 (PMC12593253; doi:10.1111/desc.70090)
Supplement: Supplementary file 1 — Supporting File 1: desc70090‐sup‐0001‐SuppMat.docx [file DESC-29-e70090-s001.docx]

**Individual differences in infants’ curiosity are linked to cognitive capacity in early childhood**

Eline R. de Boer^*^, Francesco Poli, Marlene Meyer, Rogier B. Mars, & Sabine Hunnius

* Correspondence addressed to: eline.r.deboer@gmail.com

**Supplementary Analyses**

**Supplementary Analysis S1**

In our main analysis, we examined the relation between infants’ sensitivity to information with childhood intelligence while controlling for SES using an additive model. In this model, participants’ Full Scale IQ was included as dependent variable, infants’ sensitivity to information as independent variable, and SES (indexed by the average educational level of caregiver(s)) was added as covariate.

model = bam(TIQ_IQ ~ s(LT1) + EduParents, data = data)

Here, we present these results when additionally controlling for other cognitive abilities. Specifically, we control for processing speed (SL0, representing the speed of saccadic latencies), learning performance (SL1, representing the strength of the correlation between saccadic latency and predictability of stimulus), and sustained attention (lambda0, representing looking duration to the screen)

model = bam(TIQ_IQ ~ s(LT1) + s(SL0) + s(SL1) + s(lambda0) + EduParents, data = data)

The results are presented in the supplementary table 1.

**Supplementary Table 1**

*Results of the generalized additive model relating infants’ sensitivity to information with childhood Full Scale IQ, while controlling for processing speed (SL0), learning performance (SL1) and sustained attention (lambda0).*

| Predictor | edf | F | p |
| --- | --- | --- | --- |
| Sensitivity to information gain (LT1) | 3.512 | 3.654 | 0.0097 ** |
| Processing speed (SL0) | 1 | 0.232 | 0.6318 |
| Learning performance (SL1) | 1 | 0.389 | 0.5358 |
| Sustained attention (lambda0) | 1 | 0.017 | 0.8983 |
